# The antimicrobial activity of cethromycin against *Staphylococcus aureus* and compared with erythromycin and telithromycin

**DOI:** 10.1186/s12866-023-02858-1

**Published:** 2023-04-20

**Authors:** Yuechen Hu, Lili Ouyang, Duoyun Li, Xiangbin Deng, Hongbo Xu, Zhijian Yu, Yeqing Fang, Jinxin Zheng, Zhong Chen, Haigang Zhang

**Affiliations:** 1 Department of Infectious Diseases and Shenzhen Key Lab of Endogenous Infection, Shenzhen Nanshan People’s Hospital and the 6Th Affiliated Hospital of Shenzhen University Health Science Center, Shenzhen, 518052 China; 2 Department of Critical Care Medicine and the Key Lab of Endogenous Infection, Shenzhen Nanshan People’s Hospital and the 6Th Affiliated Hospital of Shenzhen University Health Science Center, Shenzhen, 518052 China; 3 Department of Cardiology, Shenzhen Nanshan People’s Hospital and the 6Th Affiliated Hospital of Shenzhen University Health Science Center, Shenzhen, 518052 China

**Keywords:** *Staphylococcus aureus*, Cethromycin, Multilocus sequence typing, *Erm*, Macrolide-lincosamide-streptogramin B

## Abstract

**Background:**

This study aims to explore the antibacterial activity of cethromycin against *Staphylococcus aureus* (*S. aureus*), and its relationship with multilocus sequence typing (MLST), erythromycin ribosomal methylase (*erm*) genes and macrolide-lincosamide-streptogramin B (MLSB) phenotypes of *S. aureus.*

**Results:**

The minimum inhibitory concentrations (MICs) of cethromycin against 245 *S. aureus* clinical isolates ranged from 0.03125 to ≥ 8 mg/L, with the resistance of 38.8% in 121 methicillin-resistant *S. aureus* (MRSA). This study also found that cethromycin had strong antibacterial activity against *S. aureus*, with the MIC ≤ 0.5 mg/L in 55.4% of MRSA and 60.5% of methicillin-sensitive *S. aureus* (MSSA), respectively. The main MLSTs of 121 MRSA were ST239 and ST59, and the resistance of ST239 isolates to cethromycin was higher than that in ST59 isolates (*P* = 0.034)*.* The top five MLSTs of 124 MSSA were ST7, ST59, ST398, ST88 and ST120, but there was no difference in the resistance of MSSA to cethromycin between these STs. The resistance of *ermA* isolates to cethromycin was higher than that of *ermB* or *ermC* isolates in MRSA (*P* = 0.016 and 0.041, respectively), but the resistance of *ermB* or *ermC* isolates to cethromycin was higher than that of *ermA* isolates in MSSA (*P* = 0.019 and 0.026, respectively). The resistance of constitutive MLSB (cMLSB) phenotype isolates to cethromycin was higher than that of inducible MLSB (iMLSB) phenotype isolates in MRSA (*P* < 0.001) or MSSA (*P* = 0.036). The *ermA*, *ermB* and *ermC* genes was mainly found in ST239, ST59 and ST1 isolates in MRSA, respectively. Among the MSSA, the *ermC* gene was more detected in ST7, ST88 and ST120 isolates, but more *ermB* genes were detected in ST59 and ST398 isolates. The cMLSB phenotype was more common in ST239 and ST59 isolates of MRSA, and was more frequently detected in ST59, ST398, and ST120 isolates of MSSA.

**Conclusion:**

Cethromycin had strong antibacterial activity against *S. aureus*. The resistance of MRSA to cethromycin may had some clonal aggregation in ST239. The resistance of *S. aureus* carrying various *erm* genes or MLSB phenotypes to cethromycin was different.

**Supplementary Information:**

The online version contains supplementary material available at 10.1186/s12866-023-02858-1.

## Background

*Staphylococcus aureus* (*S. aureus*) is a common Gram-positive cocci, and now it is one of the leading pathogen of hospital and community acquired infections, often causing pneumonia, sepsis, skin and soft tissue infections, endocarditis, osteomyelitis, prosthetic joint infections, etc., with a high incidence rate and mortality [1]. Since British scholars discovered methicillin-resistant *S. aureus* (MRSA) in 1961, MRSA has become the main epidemic one of the multi-drug resistant bacteria [2]. Multilocus Sequence Typing (MLST) is a genotyping method that defines the sequence type by comparing the partial sequences of seven housekeeping genes of *S. aureus*, and using the specific allele combination in the standardized MLST database [3]. Therefore, it has good repeatability and accuracy, and is suitable for monitoring the epidemiology of *S. aureus* in different laboratories around the world [4, 5].

In recent years, the incidence rate of MRSA in tertiary hospitals in China has been more than 30%, ranking first among Gram-positive bacteria infections, and resistance rate of MRSA to erythromycin is as high as about 80% [6]. In 1952, the first generation of macrolide antimicrobials, represented by erythromycin, was applied to the clinic [7]. In the 1980s, the second generation of macrolide antimicrobials, which were roxithromycin, azithromycin, clarithromycin, etc., began to be widely used in clinical practice [8, 9]. However, it was also led to an increasing number of *S. aureus* resistant to macrolide antimicrobials [10]. The erythromycin ribosomal methylase (*erm*) gene family is large and often linked to other drug resistant genes, or carried by plasmids, and generally located in conjugated or nonconjugated transposons, thus play an important role in the resistance of *S. aureus* to macrolide antimicrobials [11]. Among these *erm* genes, *ermA*, *ermB*, *ermC* and *ermT* genes are most common in *S. aureus* [12]. Lincosamides and Streptogramins B are two kinds of drugs with different structures but the same action targets and similar functions as macrolide antimicrobials, thus they are called Macrolide-Lincosamide- Streptogramin B (MLSB) antimicrobials [13]. The MLSB phenotype can be divided into constitutive MLSB (cMLSB phenotype: rRNA methylase is always produced), or inducible MLSB (iMLSB phenotype: methylase is produced only when an inducing substance like erythromycin is present) [14]. U.S Clinical and Laboratory Standards Institute (CLSI) suggested that the inducible clindamycin-resistant test (D-test) should be conducted to distinguish the phenotypes of iMLSB and cMLSB [15]. Globally, *ermA* is detected in both cMLSB and iMLSB of MRSA, while *ermC* is mainly detected in iMLSB of methicillin-sensitive *S. aureus* (MSSA) [16–18].

In order to overcome the resistance of *S. aureus* to macrolide antimicrobials, the third-generation macrolides, namely new ketolide antimicrobials, such as telithromycin, solithromycin, and cethromycin, were developed by modifying the side chain of erythromycin [19]. Cethromycin is a new ketolide antibiotic being developed for use in respiratory tract infections, demonstrates more potent antibacterial effects than its predecessor telithromycin, and displays potent inhibition of both gram-positive and gram-negative respiratory pathogens [20]. In recent year, ketolide antimicrobials telithromycin, solithromycin and cethromycin were found didn’t induced erm*A* or erm*C* gene expression, while solithromycin could significantly induce *ermB* gene expression [21]. However, the antibacterial activity of cethromycin against *S. aureus* from China, and its relationship with *erm* genes is still unknown. Therefore, the purpose of this study is to explore the antibacterial activity of cethromycin against *S. aureus* from China and compared with erythromycin and telithromycin, and to investigate its relationship with MLST, *erm* genes and MLSB phenotypes of *S. aureus.*

## Results

### Antimicrobial susceptibilities of erythromycin, telithromycin and cethromycin against *S. aureus* clinical isolates

**Fig. 1:**
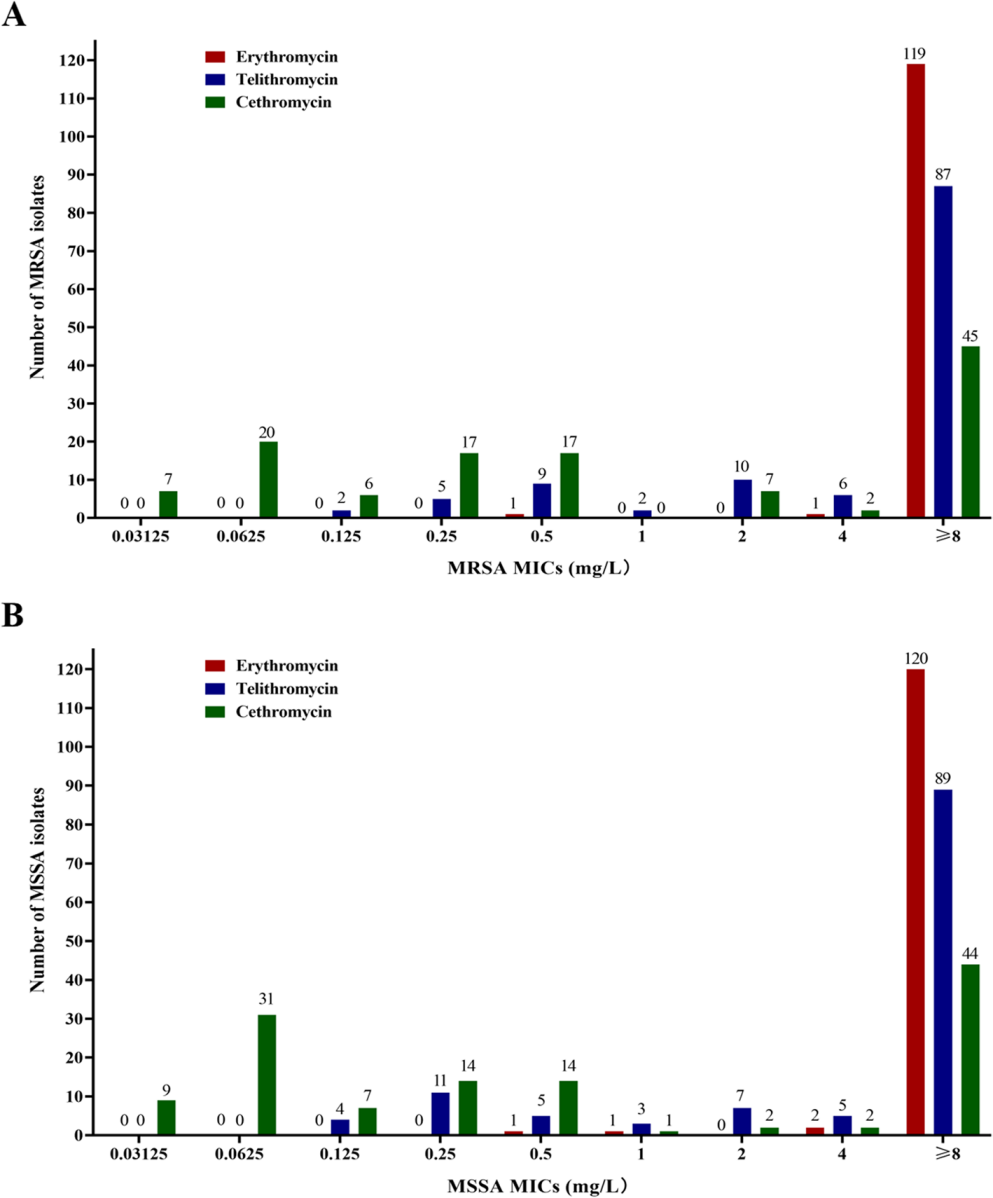
Antimicrobial susceptibilities of erythromycin, telithromycin and cethromycin against *S.aureus* Clinical isolates. **A** MICs distribution of erythromycin, telithromycin and cethromycin against MRSA. **B** MICs distribution of erythromycin, telithromycin and cethromycin against MSSA. MIC, minimum inhibitory concentration; MRSA, methicillin-resistant *Staphylococcus aureus*; MSSA, methicillin-sensitive *Staphylococcus aureus*

The minimum inhibitory concentrations (MICs) of telithromycin and cethromycin against 245 *S. aureus* clinical isolates were detected by the agar dilution method, and MICs of oxacillin (to identify MRSA or MSSA) and erythromycin were identified by the broth macrodilution method. The information of 245 *S. aureus* clinical isolates, such as isolate ID, MLSTs, isolated date, MICs, *erm* genes and MLSB phenotypes, were summarized in Table S1 and Table S2. As Fig. 1A indicated, the MICs of cethromycin against 121 MRSA ranged from 0.03125 to ≥ 8 mg/L, including 47 resistant isolates (≥ 4 mg/L), with the resistance rate of 38.8% (47/121). The resistance of MRSA to erythromycin (≥ 8 mg/L) was 98.3% (119/121) and to telithromycin (≥ 4 mg/L) was 76.9% (93/121), which was much higher than that of cethromycin (*P* < 0.001 and *P* < 0.001, respectively). This difference is also found in MSSA, that is, the resistance of MSSA to cethromycin (37.1%, 46/124) is far lower than that of erythromycin (96.8%, 120/124) and telithromycin (75.8%, 94/124), with all the *P* values < 0.001 (Fig. 1B).

Interestingly, this study also found that cethromycin had strong antibacterial activity against *S. aureus*, and its MIC ≤ 0.5 mg/L is as high as 55.4% (67/121) in MRSA and 60.5% (75/124) in MSSA, which were much higher than that of telithromycin against MRSA (13.2%, 16/121) and MSSA (16.1%, 20/124), with all the *P* values < 0.001 (Fig. 1A and B).

### Antimicrobial susceptibilities of erythromycin, telithromycin and cethromycin against different MLSTs of *S. aureus* clinical isolates

This study found that the main MLSTs of 121 MRSA were ST239 and ST59, and the resistance of ST239 isolates (51.7%) to cethromycin was higher than that in ST59 isolates (29.7%), *P* = 0.034 (Table 1). However, the resistance of ST59 isolates (97.3%) to telithromycin was higher than that in ST239 isolates (60.0%), *P* < 0.001. There was no difference in the resistance of different STs of MRSA to erythromycin. Among the 124 MSSA, the top five STs were ST7, ST59, ST398, ST88 and ST120, respectively. However, there was no difference in the resistance of MSSA to erythromycin, telithromycin or cethromycin between these STs (Table 1).

**Table 1:** Resistance of erythromycin, telithromycin and cethromycin against different MLST of *S. aureus* clinical isolates

**MLST (No.)**	**Erythromycin resistance (MIC ≥ 8 mg/L)**	**Telithromycin resistance (MIC ≥ 4 mg/L)**	*P* values^*^	**Cethromycin resistance (MIC ≥ 4 mg/L)**	***P* values**^#^
MRSA					
ST239(60)	59 (98.3%)	36(60.0%)		31(51.7%)	
ST59(37)	37(100.0%)	36(97.3%)	< 0.001^**a**^	11(29.7%)	0.034^**a**^
ST1(7)	7(100.0%)	5(71.4%)		0(0.0%)	
NT(5)	5(100.0%)	4(80.0%)		2(40.0%)	
Others(12)	12(100.0%)	12(100.0%)		3(25.0%)	
MSSA					
ST7(27)	27(100.0%)	23(85.2%)		10(37.0%)	
ST59(20)	20(100.0%)	19(95.0%)		12(65.0%)	
ST398(12)	12(100.0%)	10(93.3%)		5(41.7%)	
ST88(7)	7(100.0%)	4(57.1%)		2(28.6%)	
ST120(6)	5(83.3%)	4(66.7%)		2(33.3%)	
NT(11)	11(100.0%)	8(72.7%)		4(36.4%)	
Others(41)	40(97.6%)	24(58.5%)		11(26.8%)	

*MLST* Multilocus sequence typing, *MIC* Minimum inhibitory concentration, *MRSA* Methicillin-resistant *Staphylococcus aureus*, *MSSA* Methicillin-sensitive *Staphylococcus aureus*, *NT* Not detected, The data was reported as number (percentage, %) and was compared using the chi-square test

^*^P values for telithromycin resistance group

^#^*P* values for cethromycin resistance group

^**a**^ST59 vs ST239

### Antimicrobial susceptibilities of erythromycin, telithromycin and cethromycin against *S. aureus* clinical isolates with *erm* genes or MLSB phenotypes

Among the 121 MRSA, 116 isolates were *erm* genes positive (1 isolate was both *ermA* and *ermB* positive; 1 isolate was both *ermB* and *ermC* positive), and *erm* gene was not detected in 5 isolates. The resistance of *ermA* MRSA to cethromycin was higher than that of *ermB* or *ermC* isolates (*P* = 0.016 and *P* = 0.041, respectively). However, the resistance of *ermB* or *ermC* MRSA to telithromycin was higher than that of *ermA* isolates (*P* < 0.001 and *P* = 0.048, respectively). This study also found that the resistance of cMLSB phenotype isolates to cethromycin or telithromycin in MRSA was significantly higher than that of iMLSB phenotype isolates, *P* < 0.001 and *P* = 0.025, respectively (Table 2).

**Table 2:** Resistance of erythromycin, telithromycin and cethromycin against *S. aureus* clinical isolates with *erm* genes or MLSB phenotypes

***erm* or MLSB (No.)**	**Erythromycin resistance (MIC ≥ 8 mg/L)**	**Telithromycin resistance (MIC ≥ 4 mg/L)**	*P* values^*^	**Cethromycin resistance (MIC ≥ 4 mg/L)**	***P* values**^#^
MRSA					
*ermA*(56)	56 (100.0%)	32 (57.1%)		29 (51.8%)	
*ermB*(46)	46 (100.0%)	43 (93.5%)	< 0.001^**a**^	13 (28.3%)	0.016^**a**^
*ermC*(14)	13 (92.9%)	12 (85.7%)	0.048^**b**^	3 (21.4%)	0.041^**b**^
cMLSB (104)	103 (99.0%)	84 (80.8%)	0.025^**c**^	47 (45.2%)	< 0.001^**c**^
iMLSB (17)	17 (100.0%)	9 (52.9%)		0 (0.0%)	
MSSA					
*ermA*(9)	8 (88.9%)	1 (11.1%)		0 (0.0%)	
*ermB* (42)	42 (100.0%)	37 (88.1%)	< 0.001^**a**^	18 (42.9%)	0.019^**a**^
*ermC* (72)	71 (98.6%)	54 (75.0%)	< 0.001^**b**^	27 (37.5%)	0.026^**b**^
cMLSB (55)	54 (98.2%)	49 (89.1%)	0.002^**c**^	26 (47.3%)	0.036^**c**^
iMLSB (69)	68 (98.6%)	45 (65.2%)		20 (29.0%)	

*MLSB* Macrolide-lincosamide-streptogramin B, *cMLSB* Constitutive MLSB; iMLSB, Inducible MLSB, *MIC* Minimum inhibitory concentration, *MRSA* Methicillin-resistant *Staphylococcus aureus*, *MSSA* Methicillin-sensitive *Staphylococcus aureus*, The data was reported as number (percentage, %) and was compared using the chi-square test or Fisher’s exact test

^*^P values for telithromycin resistance group

^#^*P* values for cethromycin resistance group

^**a**^*ermB* vs *ermA*

^**b**^*ermC* vs *ermA*

^c^cMLSB vs iMLSB

The present research indicated that among the 124 MSSA, 121 isolates were *erm* genes positive (2 isolates were both *ermB* and *ermC* positive), and *erm* gene was not detected in 3 isolates. The resistance of *ermB* or *ermC* MSSA to cethromycin was higher than that of *ermA* isolates (*P* = 0.019 and *P* = 0.026, respectively). This similar difference was also found in the telithromycin resistant MSSA isolates, that is the resistance of *ermB* or *ermC* MSSA isolates to telithromycin was higher than that of *ermA* isolates, with all the *P* values < 0.001. This study also found that, similar to the above results in MRSA isolates, the resistance of cMLSB phenotype MSSA isolates to cethromycin or telithromycin was higher than that of iMLSB phenotype isolates, *P* = 0.036 and *P* = 0.002, respectively (Table 2).

### Distribution of *erm* genes or MLSB phenotypes in different MLST of *S. aureus*

**Fig. 2:**
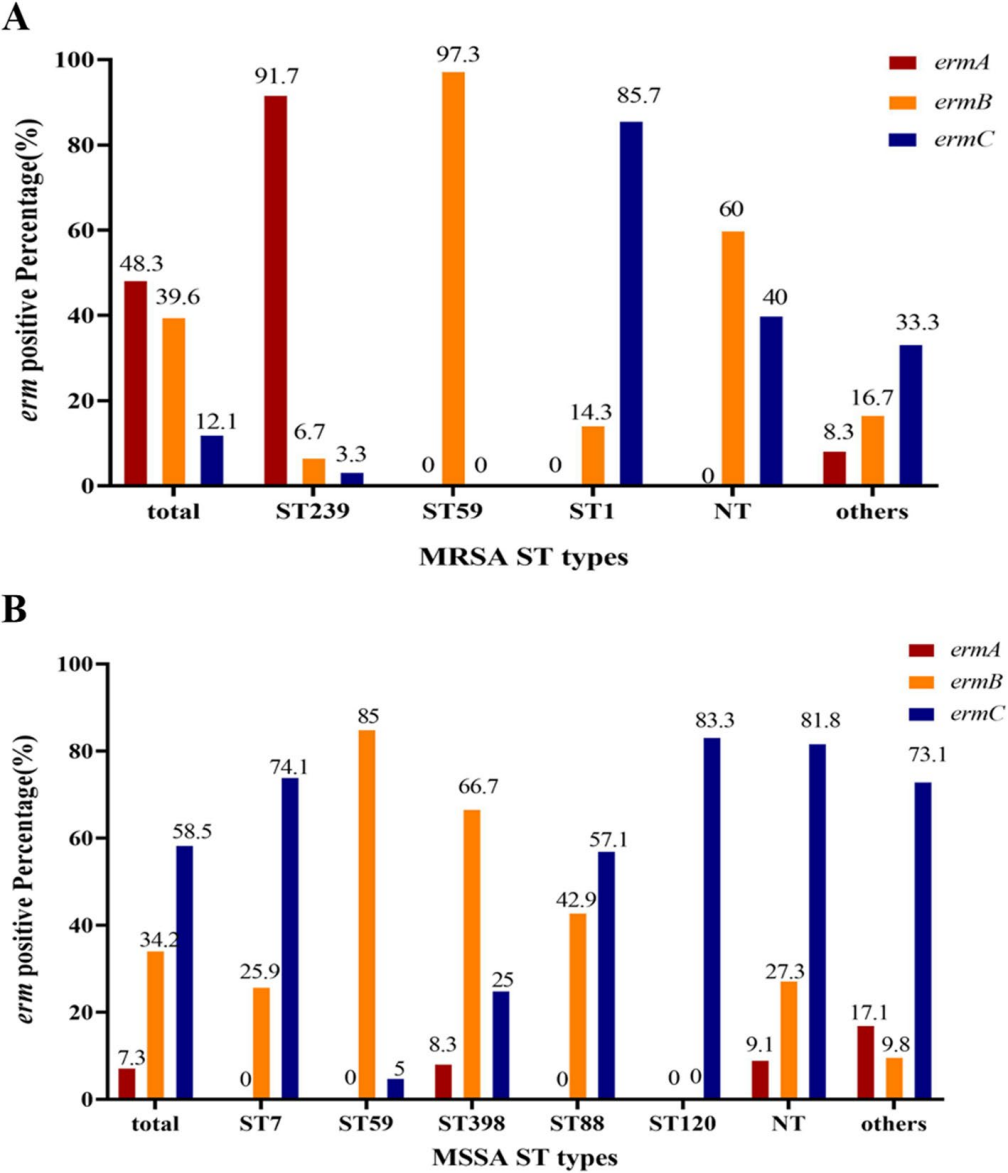
Distribution of *erm* genes in different MLST of MRSA (**A**) or MSSA (**B**)*.* MLST, Multilocus Sequence Typing; NT*,* not detected; MRSA, methicillin-resistant *Staphylococcus aureus*; MSSA, methicillin-sensitive *Staphylococcus aureus*

In 121 MRSA, *ermA* was mainly found in ST239 isolates (91.7%), *ermB* was as high as 97.3% in ST59 isolates, and *ermC* was also found mainly in ST1 isolates (85.7%). The *erm* genes showed more obvious ST aggregation in 124 MSSA, and *ermC* was more detected in ST7 (74.1%), ST88 (57.1%) and ST120 (83.3%) isolates. However, more *ermB* were detected in ST59 (85.0%) and ST398 (66.7%) isolates (Fig. 2).

**Fig. 3:**
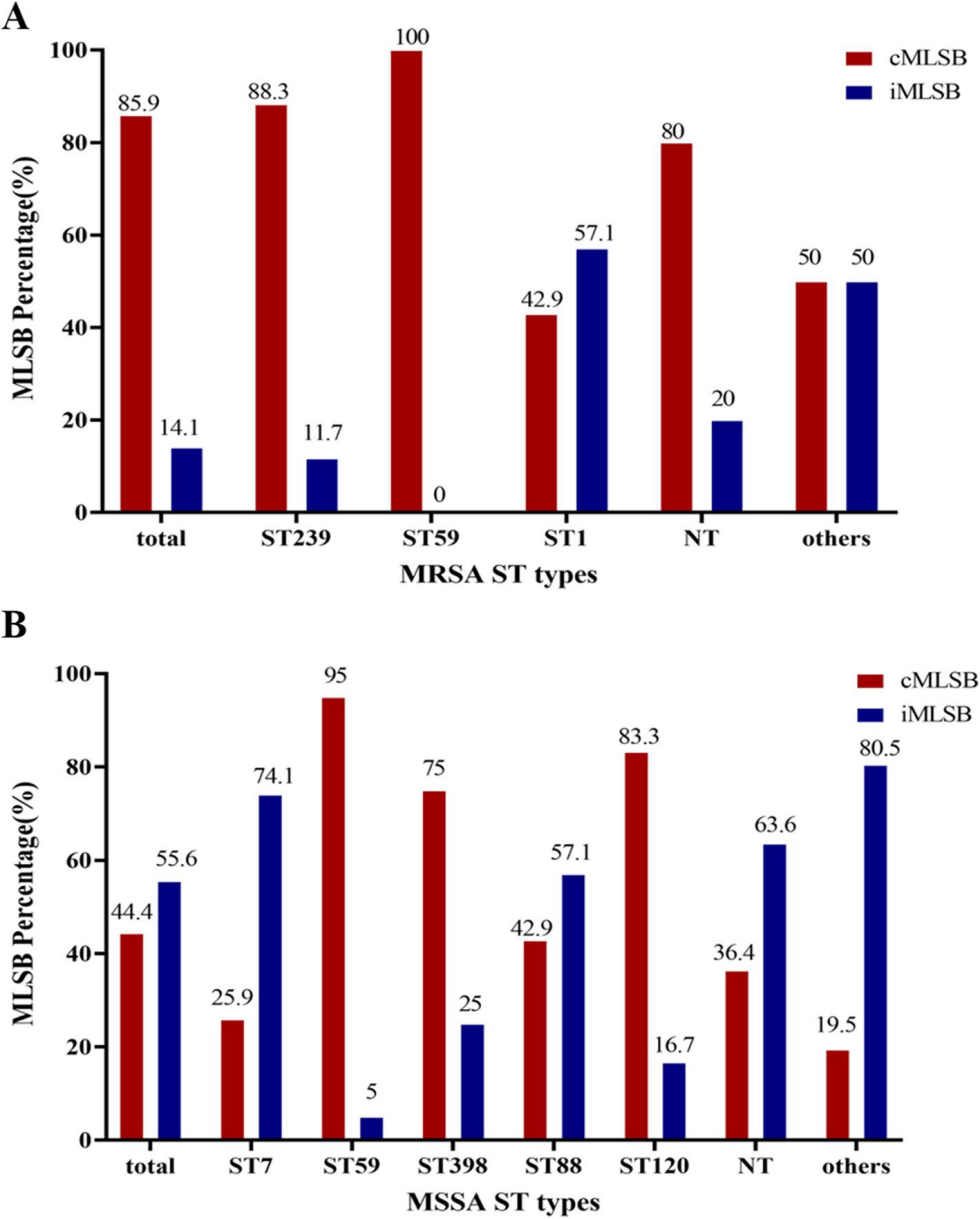
Distribution of MLSB phenotypes in different MLST of MRSA (**A**) or MSSA (**B**)***.*** MLSB, Macrolide-Lincosamide-Streptogramin B; cMLSB, constitutive MLSB; iMLSB, inducible MLSB; MLST, Multilocus Sequence Typing; NT*,* not detected; MRSA, methicillin-resistant *Staphylococcus aureus*; MSSA, methicillin-sensitive *Staphylococcusaureus*

The cMLSB phenotype was more common in ST239 (88.3%) and ST59 (100.0%) isolates of MRSA, and also was more frequently detected in ST59 (95.0%), ST398 (75.0%), and ST120 (83.3%) isolates of MSSA (Fig. 3).

### Distribution of MLSB phenotypes in *S. aureus *with different *erm* genes

**Fig. 4:**
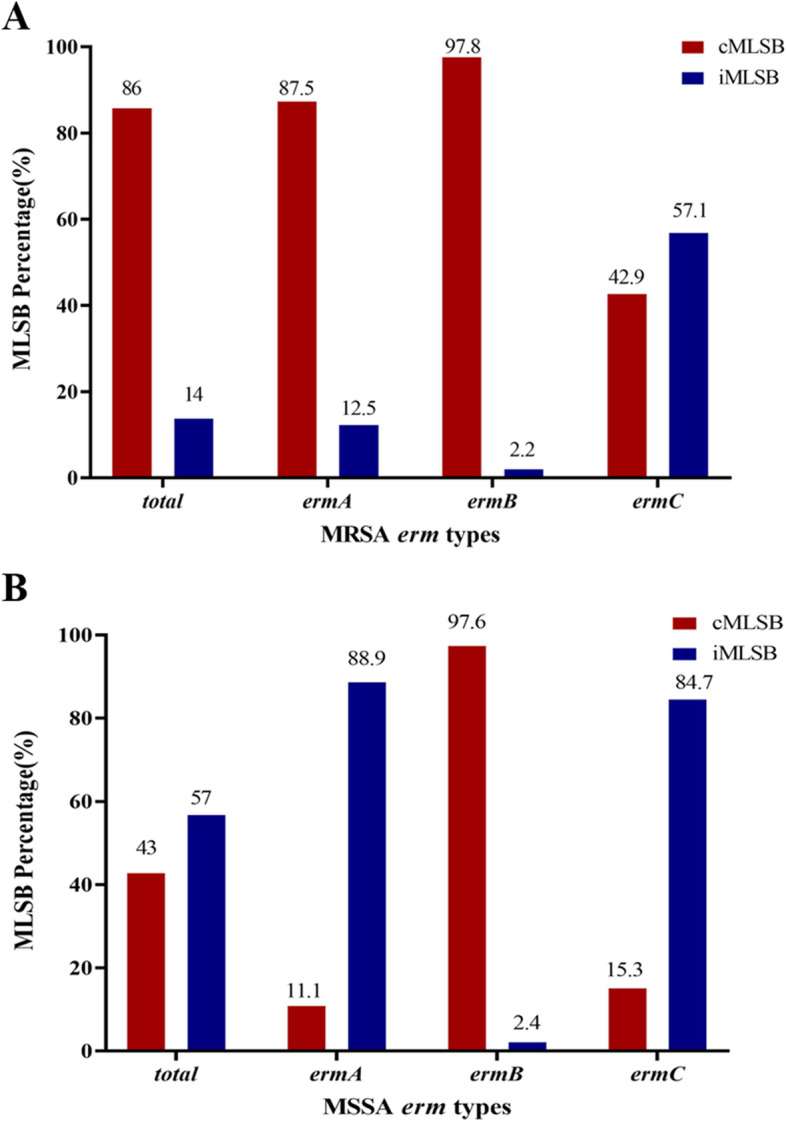
Distribution of MLSB phenotypes in different *erm* genes of MRSA (**A**) or MSSA (**B**)***.*** MLSB, Macrolide-Lincosamide-Streptogramin B; cMLSB, constitutive MLSB; iMLSB, inducible MLSB; MRSA, methicillin-resistant *Staphylococcus aureus*; MSSA, methicillin-sensitive *Staphylococcus aureus*

The cMLSB phenotype was more common in *ermA* (87.5%) or *ermB* (97.8%) isolates of the 121 MRSA. While in the 124 MSSA, the cMLSB phenotype was only more frequently detected in *ermB* (97.6%) isolates, and the iMLSB phenotype was more determined in *ermA* (88.9%) and *ermC* (84.7%) isolates (Fig. 4).

## Discussion

Cethromycin, a new ketolide, has a similar mechanism with telithromycin but with an apparently better safety profile, indicates excellent activities against selected gram-positive, gram-negative, atypical bacteria, and even designed for tularemia, plague, and anthrax prophylaxis [22]. Recently it has been found that telithromycin induced liver toxicity, thus the United States Food and Drug Agency (US FDA) has restricted the indications of telithromycin. Interestingly, unlike telithromycin, cethromycin does not induce adverse hepatotoxic reactions and has good pharmacokinetic properties [23]. This study indicated that the antibacterial activity of cethromycin against 121 MRSA and 124 MSSA was significantly stronger than that of erythromycin or telithromycin. In the present study, the resistance of *S. aureus* to erythromycin was more than 99%, which was higher than the average level of Class III hospitals in China [6]. This study found that the resistance of 121 MRSA and 124 MSSA to cethromycin reached 38.8% and 37.1% respectively, which were higher than the results of Luna VA et al. [24]. In the study of Luna VA et al., the resistance of 170 MRSA cloned from USA300 in the United States to erythromycin was 90.6%, and no isolates resistant to cethromycin were found.

The main MLSTs of MRSA in this study were ST239 and ST59, which were consistent with most reports from MRSA epidemic isolates. *S. aureus* ST239 clone was found in Brazil for the first time [25]. Since then, it has been widely popular worldwide. ST239 clone is the main epidemic type in the world at present, and also the main epidemic strain causing HA-MRSA (healthcare-associated MRSA) in China [26, 27]. ST59 has always been considered the most common clone of CA-MRSA (community-associated MRSA) in China [27]. The MLSTs of MSSA detected in this study was relatively scattered. Previous studies have found that ST398, ST5, ST88 and ST7 are the more common epidemic MLSTs of MSSA in China [28, 29]. This study found that MRSA of ST239 and ST59 had different resistance rates to cethromycin, suggesting that these MRSA with different *erm* genes and cMLSB/iMLSB phenotypes.

This study demonstrated that *ermA* and *ermB* in MRSA was significantly related to the main MLSTs of ST239 and ST59, respectively, and they were mainly cMLSB phenotype, suggesting that highly homologous mobile elements or plasmids integrating *ermA* or *ermB* might transfer and spread in MRSA dominant phenotypes, leading to the occurrence of cMLSB phenotype. However, in MSSA, MLSTs were relatively scattered, the proportion of cMLSB and iMLSB is close, and *ermB* and *ermC* were mainly detected. The iMLSB phenotype was the main phenotype in *ermA* and *ermC* isolates, and the cMLSB phenotype was the main phenotype in *ermB* isolates. T Otsuka et al. found that in MRSA, the cMLSB phenotype accounted for 61.3%, while in MSSA, only 1.3%, mainly *ermA* gene; the iMLSB phenotype was 38.7% in MRSA and 94.0% in MSSA, with *ermC* as the main *erm* gene [30]. The results of this study were similar to those of T Otsuka et al., which also detected *erm* gene mainly with *ermA,* and MLSB phenotype mainly with cMLSB in MRSA.

Previous studies have shown that the iMLSB phenotype in Staphylococcus may be mediated by *ermA* or *ermC* [31, 32]. This study shown that most of the MLSTs in MSSA were related to *ermC*, and MSSA with *ermC* was dominated by iMLSB phenotype. In this study, there were five isolates of ST5 in all *S. aureus*, all of which had *ermC* gene and were all with iMLSB phenotype. However, in Ilczyszyn et al.’s research, CC5 (ST5) is the main popular clone of *S. aureus*, with *ermA* gene detected mostly [33]. In addition, Japanese researchers conducted comparative genomic analysis of a multi-drug resistant CA-MRSA-ST59-SCCmec V strain, and found that the *ermB* detected was integrated on a 21 kb mobile element containing a chromosome with a complex transposon Tn551-Tn5404 [34]. In this study, whether *ermB* in the dominant type ST59 of *S. aureus* was also integrated on the chromosome for transfer and transmission needs to be verified by further experiments. In addition, it should be noted that MRSA and MSSA in this study each had one isolate carrying *ermC* gene, but this isolate was sensitive to erythromycin. This indicated that the *ermC* carried by *S. aureus* may not be resistant to erythromycin, and the specific mechanism needs to be further explored.

## Conclusion

This study found that cethromycin had strong antibacterial activity against *S. aureus*, and had antibacterial activity against erythromycin resistant *S. aureus*. The resistance of MRSA to cethromycin may had some clonal aggregation in ST239. The resistance of *ermA* MRSA to cethromycin was higher than that of *ermB* or *ermC* isolates, but the resistance of *ermB* or *ermC* MSSA to cethromycin was higher than that of *ermA* isolates. The resistance of cMLSB phenotype isolates to cethromycin in MRSA or MSSA was significantly higher than that of iMLSB phenotype isolates.

## Methods

### Bacterial isolates and chemicals

A total of 245 *S. aureus* clinical isolates [Isolated from sputum (79), throat swabs (46), blood (39), pus (35), catheters (24), pleural effusion (14), and cerebrospinal fluid (8)] were retrospectively collected from the inpatients of Shenzhen Nanshan People’s Hospital (which includes 1200 beds), Shenzhen University in China, from 1 January 2012 to 31 December 2015. All clinical isolates were identified with the Phoenix 100 automated microbiology system (BD, Franklin Lakes, NJ, USA) and matrix-assisted laser desorption ionization time-of-flight mass spectrometry (IVD MALDI Biotyper, Germany). After being identified as *S. aureus*, the monoclone of *S. aureus* was picked up and cultured, then the cultures were added in glycerol with the final concentration of 30%, finally were save in—80℃ refrigerator.

*S. aureus* isolates were grown in tryptic soy broth (TSB) at 37 °C with shaking of 180 rpm unless otherwise stated. For the antimicrobial susceptibility test, isolates were grown in cation-adjusted Mueller–Hinton broth (CAMHB) at 37 °C with shaking.

Oxacillin sodium (catalogue no. HY-B0465), erythromycin (catalogue no. HY-B0220), telithromycin (catalogue no. HY-A0062), cethromycin (catalogue no. HY-19655) were purchased from MedChemExpress (MCE, Shanghai, China).

### Antimicrobial susceptibility testing

The minimum inhibitory concentrations (MICs) of oxacillin and erythromycin against *S. aureus* clinical isolates were determined by the broth macrodilution method, MICs of telithromycin and cethromycin against *S. aureus* clinical isolates were determined by the agar dilution method, according to the Clinical and Laboratory Standards Institute guidelines (CLSI-M100-S27). All experiments were performed in triplicate. The antimicrobial susceptibility breakpoints of oxacillin, erythromycin, and telithromycin were confirmed based on CLSI-M100-S27. As no CLSI susceptibility breakpoints for cethromycin were recommended against *S. aureus*, thus the susceptibility breakpoints for telithromycin against *S. aureus* in CLSI-M100-S27 were used for reference in this study, that is, the resistance of cethromycin against *S. aureus* were ≥ 4 mg/L.

### MLST analysis

The genomic DNA of *S. aureus* clinical isolates was extracted with a commercial DNA extraction kit (Dalian Bio Engineering China Co., Ltd.). MLST analysis was conducted according to the previous study [35]. In short, seven housekeeping genes (*arcC*, *aroE*, *glpF*, *gmk*, *pta*, *tpi*, *yqiL*) of *S. aureus* were PCR amplified and sequenced, and PCR amplification was performed in a total volume of 50 μl, containing 2 × PCR Master Mix (Tiangen Biotech Beijing Co., Ltd, Beijing, China), 0.5 mM of each primer, and 1 µl template DNA. The cycling conditions were as follows: 94 °C for 5 min; followed by 30 cycles at 94 °C for 45 s, 45 °C for 30 s, 72 °C for 60 s; and a final 5 min extension step at 72 °C. Each PCR set included a no-template control and a positive control. Alleles and sequence types (STs) were assigned by the MLST database (http://saureus.mlst.net/Staphylococcus* aureus*.html). All PCR primers used for MLST analysis were listed in Table S3.

### Detection of *erm* genes

The macrolide antimicrobials ribosomal methylase genes (*erm* genes), *ermA*, *ermB* and *ermC*, were detected by PCR based on the previous study [36, 37]. The genomic DNA of *S. aureus* clinical isolates was extracted as above mentioned. The PCR reaction system was as described above, and only the PCR primers were different. The cycling conditions were as follows: 93 °C for 2 min; followed by 30 cycles at 93 °C for 30 s, 55 °C for 30 s, 72 °C for 60 s; and a final 5 min extension step at 72 °C. Each PCR set included a no-template control and a positive control. All PCR primers used for the detection of *erm* genes were listed in Table S4.

### D-zone test

The inducible clindamycin-resistant test (D-zone test) conducted to determine the phenotypes of iMLSB and cMLSB according to the previous study [38]. The erythromycin and clindamycin susceptibility test discs were purchased from Sigma Aldrich (shanghai, China). After 16 to 18 h of incubation, the D-zone test results were assessed by transmitted or reflected light and interpreted according to the Clinical and Laboratory Standards Institute (CLSI) guidelines.

### Statistical analysis

The data of the present study was reported as a number (percentage) and was compared using the chi-square test or Fisher’s exact test. *P* values < 0.05 were regarded as statistically significant. All data were analyzed using the statistical software SPSS (version 20.0; SPSS, Chicago, IL, USA).

## Supplementary Information

**Additional file 1:** **Table S1**. The supplementary information of 121 MRSA in this study. **Table S2**. The supplementary information of 124 MSSA in this study. **Table S3**. PCR primers used for *S. aureus* MLST gene diversity determination. **Table S4**. PCR primers used for *S. aureus* *erm* genes detection.

## Data Availability

All data generated or analyzed during this study are included in this published article [and its supplementary information files].

## Abbreviations

*Erm*: Erythromycin ribosomal methylase
MIC: Minimum inhibitory concentration
MLSB: Macrolide-lincosamide-streptogramin B
cMLSB: Constitutive MLSB
iMLSB: Inducible MLSB
MLST: Multilocus sequence typing
MRSA: Methicillin-resistant *Staphylococcus aureus*
MSSA: Methicillin-sensitive *Staphylococcus aureus*
