# Supplementary material for: The antimicrobial activity of cethromycin against Staphylococcus aureus and compared with erythromycin and telithromycin
Source: BMC Microbiol. 2023 Apr 20;23:109. doi: 10.1186/s12866-023-02858-1 (PMC10116812; doi:10.1186/s12866-023-02858-1)
Supplement: Supplementary file 1 — Additional file 1: Table S1. The supplementary information of 121 MRSA in this study. Table S2. The supplementary information of 124 MSSA in this study. Table S3. PCR primers used for S. aureus MLST gene diversity determination. Table S4. PCR primers used for S. aureus erm genes detection. [file 12866_2023_2858_MOESM1_ESM.docx]

**Supplementary Tables:**

**Table S1**. The supplementary information of 121 MRSA in this study.

| **Isolates-ID** | **Isolated date** | **MLSTs** |  | **MIC (mg/L)** | | |  | ***ermA*** | ***ermB*** | ***ermC*** | **D-test** |
| --- | --- | --- | --- | --- | --- | --- | --- | --- | --- | --- | --- |
|  |  |  |  | **Ery** | **Tel** | **Cet** |  |  |  |  |  |
| 13SA006631 | 2013-7-3 | NT |  | ≥ 8 | >16 | >16 |  |  |  | ＋ | C |
| 13SA005991 | 2013-9-8 | NT |  | ≥ 8 | >16 | >16 |  |  |  | ＋ | C |
| 13SA002211 | 2013-3-17 | NT |  | ≥ 8 | >16 | 0.5 |  |  | ＋ |  | C |
| 13SA007021 | 2013-11-1 | NT |  | ≥ 8 | 0.5 | 0.0625 |  |  | ＋ |  | C |
| 13SA005351 | 2013-9-2 | NT |  | ≥ 8 | >16 | 0.03125 |  |  | ＋ |  | I |
| 15SA001911 | 2015-6-8 | ST1 |  | ≥ 8 | >16 | 0.0625 |  |  |  | ＋ | C |
| 13SA005521 | 2013-9-3 | ST1 |  | ≥ 8 | >16 | 0.125 |  |  |  | ＋ | C |
| 13SA004741 | 2013-3-29 | ST1 |  | ≥ 8 | >16 | 0.0625 |  |  | ＋ |  | C |
| 13SA004001 | 2013-7-5 | ST1 |  | ≥ 8 | 0.5 | 0.03125 |  |  |  | ＋ | I |
| 14SA000851 | 2014-2-6 | ST1 |  | ≥ 8 | >16 | 0.0625 |  |  |  | ＋ | I |
| 13SA006531 | 2013-10-5 | ST1 |  | ≥ 8 | 0.5 | 0.0625 |  |  |  | ＋ | I |
| 13SA006001 | 2013-9-8 | ST1 |  | ≥ 8 | >16 | 0.0625 |  |  |  | ＋ | I |
| 13SA007891 | 2013-12-1 | ST1821 |  | ≥ 8 | >16 | >16 |  |  | ＋ |  | C |
| 13SA001081 | 2013-3-8 | ST188 |  | ≥ 8 | 4 | 0.25 |  |  |  |  | C |
| 12TA000511 | 2012-6-6 | ST188 |  | ≥ 8 | >16 | 0.0625 |  |  |  | ＋ | I |
| 13SA009571 | 2013-7-2 | ST188 |  | ≥ 8 | >16 | 0.03125 |  |  |  |  | I |
| 12TA000041 | 2012-7-23 | ST237 |  | ≥ 8 | >16 | 0.5 |  |  |  |  | C |
| 12TA000261 | 2012-7-23 | ST238 |  | ≥ 8 | >16 | 0.25 |  |  |  |  | C |
| 13SA007991 | 2013-7-3 | ST239 |  | ≥ 8 | 8 | >16 |  |  | ＋ | ＋ | C |
| 13SA003561 | 2013-3-17 | ST239 |  | 0.5 | >16 | 0.0625 |  |  |  | ＋ | C |
| 13SA009351 | 2013-7-2 | ST239 |  | ≥ 8 | 2 | 0.25 |  | ＋ | ＋ |  | C |
| 13SA001491 | 2013-3-17 | ST239 |  | ≥ 8 | >16 | 0.25 |  |  | ＋ |  | C |
| 13SA006351 | 2013-7-3 | ST239 |  | ≥ 8 | >16 | >16 |  |  | ＋ |  | C |
| 13SA005641 | 2013-7-3 | ST239 |  | ≥ 8 | 0.125 | 0.03125 |  | ＋ |  |  | C |
| 13SA008171 | 2013-7-2 | ST239 |  | ≥ 8 | 4 | 0.03125 |  | ＋ |  |  | C |
| 13SA000841 | 2013-3-8 | ST239 |  | ≥ 8 | 2 | 0.0625 |  | ＋ |  |  | C |
| 13SA007761 | 2013-7-2 | ST239 |  | ≥ 8 | 2 | 0.0625 |  | ＋ |  |  | C |
| 12TA000591 | 2012-7-23 | ST239 |  | ≥ 8 | 0.25 | 0.0625 |  | ＋ |  |  | C |
| 13SA008501 | 2013-7-2 | ST239 |  | ≥ 8 | 2 | 0.125 |  | ＋ |  |  | C |
| 13SA008771 | 2013-7-2 | ST239 |  | ≥ 8 | 2 | 0.125 |  | ＋ |  |  | C |
| 13SA002021 | 2013-3-17 | ST239 |  | ≥ 8 | 8 | 0.25 |  | ＋ |  |  | C |
| 13SA001911 | 2013-3-17 | ST239 |  | ≥ 8 | >16 | 0.25 |  | ＋ |  |  | C |
| 13SA002571 | 2013-3-17 | ST239 |  | ≥ 8 | 4 | 0.25 |  | ＋ |  |  | C |
| 13SA000911 | 2013-3-8 | ST239 |  | ≥ 8 | 0.25 | 0.25 |  | ＋ |  |  | C |
| 13SA002581 | 2013-3-17 | ST239 |  | ≥ 8 | 8 | 0.25 |  | ＋ |  |  | C |
| 13SA000571 | 2013-3-8 | ST239 |  | ≥ 8 | 2 | 0.25 |  | ＋ |  |  | C |
| 13SA000641 | 2013-3-8 | ST239 |  | ≥ 8 | 2 | 0.5 |  | ＋ |  |  | C |
| 13SA004151 | 2013-3-17 | ST239 |  | ≥ 8 | 8 | 0.5 |  | ＋ |  |  | C |
| 13SA001001 | 2013-3-8 | ST239 |  | ≥ 8 | 16 | 2 |  | ＋ |  |  | C |
| 13SA000501 | 2013-3-8 | ST239 |  | 4 | 4 | 2 |  | ＋ |  |  | C |
| 13SA001041 | 2013-3-8 | ST239 |  | ≥ 8 | >16 | 2 |  | ＋ |  |  | C |
| 13SA003201 | 2013-3-17 | ST239 |  | ≥ 8 | >16 | 2 |  | ＋ |  |  | C |
| 13SA003321 | 2013-3-17 | ST239 |  | ≥ 8 | >16 | 8 |  | ＋ |  |  | C |
| 13SA009141 | 2013-7-2 | ST239 |  | ≥ 8 | 16 | >16 |  | ＋ |  |  | C |
| 13SA001701 | 2013-3-17 | ST239 |  | ≥ 8 | 4 | >16 |  | ＋ |  |  | C |
| 13SA003581 | 2013-3-17 | ST239 |  | ≥ 8 | 16 | >16 |  | ＋ |  |  | C |
| 13SA006371 | 2013-7-3 | ST239 |  | ≥ 8 | >16 | >16 |  | ＋ |  |  | C |
| 13SA005831 | 2013-7-3 | ST239 |  | ≥ 8 | 8 | >16 |  | ＋ |  |  | C |
| 13SA006231 | 2013-7-3 | ST239 |  | ≥ 8 | 2 | >16 |  | ＋ |  |  | C |
| 13SA002751 | 2013-3-17 | ST239 |  | ≥ 8 | 8 | >16 |  | ＋ |  |  | C |
| 13SA004361 | 2013-3-17 | ST239 |  | ≥ 8 | >16 | >16 |  | ＋ |  |  | C |
| 13SA005881 | 2013-7-3 | ST239 |  | ≥ 8 | 16 | >16 |  | ＋ |  |  | C |
| 13SA006841 | 2013-7-3 | ST239 |  | ≥ 8 | 1 | >16 |  | ＋ |  |  | C |
| 13SA003671 | 2013-3-17 | ST239 |  | ≥ 8 | 0.5 | >16 |  | ＋ |  |  | C |
| 13SA005901 | 2013-7-3 | ST239 |  | ≥ 8 | 0.5 | >16 |  | ＋ |  |  | C |
| 13SA009201 | 2013-7-2 | ST239 |  | ≥ 8 | 1 | >16 |  | ＋ |  |  | C |
| 13SA008241 | 2013-7-3 | ST239 |  | ≥ 8 | >16 | >16 |  | ＋ |  |  | C |
| 13SA000661 | 2013-3-8 | ST239 |  | ≥ 8 | 0.25 | >16 |  | ＋ |  |  | C |
| 13SA009091 | 2013-7-2 | ST239 |  | ≥ 8 | >16 | >16 |  | ＋ |  |  | C |
| 13SA002121 | 2013-3-17 | ST239 |  | ≥ 8 | >16 | >16 |  | ＋ |  |  | C |
| 13SA006261 | 2013-7-3 | ST239 |  | ≥ 8 | >16 | >16 |  | ＋ |  |  | C |
| 13SA006641 | 2013-7-3 | ST239 |  | ≥ 8 | >16 | >16 |  | ＋ |  |  | C |
| 13SA005491 | 2013-7-3 | ST239 |  | ≥ 8 | >16 | >16 |  | ＋ |  |  | C |
| 13SA002981 | 2013-3-17 | ST239 |  | ≥ 8 | 0.25 | >16 |  | ＋ |  |  | C |
| 13SA002961 | 2013-3-17 | ST239 |  | ≥ 8 | 4 | >16 |  | ＋ |  |  | C |
| 13SA002991 | 2013-3-17 | ST239 |  | ≥ 8 | >16 | >16 |  | ＋ |  |  | C |
| 13SA007291 | 2013-7-2 | ST239 |  | ≥ 8 | >16 | >16 |  | ＋ |  |  | C |
| 13SA005181 | 2013-7-3 | ST239 |  | ≥ 8 | 0.25 | >16 |  | ＋ |  |  | C |
| 13SA009391 | 2013-7-2 | ST239 |  | ≥ 8 | >16 | >16 |  | ＋ |  |  | C |
| 13SA004711 | 2013-3-25 | ST239 |  | ≥ 8 | >16 | >16 |  | ＋ |  |  | C |
| 13SA007921 | 2013-7-3 | ST239 |  | ≥ 8 | >16 | >16 |  |  |  |  | C |
| 13SA002291 | 2013-3-17 | ST239 |  | ≥ 8 | >16 | 0.0625 |  | ＋ |  |  | I |
| 13SA004281 | 2013-3-17 | ST239 |  | ≥ 8 | 2 | 0.125 |  | ＋ |  |  | I |
| 13SA005271 | 2013-9-1 | ST239 |  | ≥ 8 | 0.5 | 0.0625 |  | ＋ |  |  | I |
| 13SA003501 | 2013-9-3 | ST239 |  | ≥ 8 | 0.125 | 0.0625 |  | ＋ |  |  | I |
| 13SA008781 | 2013-2-3 | ST239 |  | ≥ 8 | 0.5 | 0.0625 |  | ＋ |  |  | I |
| 13SA009381 | 2013-8-4 | ST239 |  | ≥ 8 | 0.5 | 0.0625 |  | ＋ |  |  | I |
| 13SA009801 | 2013-3-9 | ST239 |  | ≥ 8 | 0.5 | 0.0625 |  | ＋ |  |  | I |
| 14SA003011 | 2014-5-4 | ST2631 |  | ≥ 8 | >16 | 0.03125 |  |  |  | ＋ | I |
| 12TA000131 | 2012-7-4 | ST338 |  | ≥ 8 | >16 | 0.5 |  |  | ＋ |  | C |
| 13SA002631 | 2013-3-17 | ST455 |  | ≥ 8 | 16 | >16 |  |  |  |  | C |
| 13SA008391 | 2013-6-9 | ST5 |  | ≥ 8 | >16 | 0.0625 |  |  |  | ＋ | I |
| 13SA008311 | 2013-7-3 | ST59 |  | ≥ 8 | >16 | 0.125 |  |  | ＋ |  | C |
| 13SA003611 | 2013-3-17 | ST59 |  | ≥ 8 | >16 | 0.125 |  |  | ＋ |  | C |
| 13SA008921 | 2013-7-2 | ST59 |  | ≥ 8 | >16 | 0.25 |  |  | ＋ |  | C |
| 13SA006961 | 2013-7-3 | ST59 |  | ≥ 8 | >16 | 0.25 |  |  | ＋ |  | C |
| 13SA006851 | 2013-7-3 | ST59 |  | ≥ 8 | >16 | 0.25 |  |  | ＋ |  | C |
| 13SA010131 | 2013-7-3 | ST59 |  | ≥ 8 | 16 | 0.25 |  |  | ＋ |  | C |
| 13SA000951 | 2013-3-8 | ST59 |  | ≥ 8 | >16 | 0.5 |  |  | ＋ |  | C |
| 13SA003961 | 2013-3-17 | ST59 |  | ≥ 8 | >16 | 0.5 |  |  | ＋ |  | C |
| 13SA008131 | 2013-7-2 | ST59 |  | ≥ 8 | >16 | 0.5 |  |  | ＋ |  | C |
| 13SA001841 | 2013-3-17 | ST59 |  | ≥ 8 | >16 | 0.5 |  |  | ＋ |  | C |
| 15SA000051 | 2015-1-27 | ST59 |  | ≥ 8 | >16 | 0.5 |  |  | ＋ |  | C |
| 15SA000401 | 2015-4-6 | ST59 |  | ≥ 8 | >16 | 0.5 |  |  | ＋ |  | C |
| 12TA000481 | 2013-1-23 | ST59 |  | ≥ 8 | >16 | 2 |  |  | ＋ |  | C |
| 13SA002341 | 2013-3-17 | ST59 |  | ≥ 8 | >16 | >16 |  |  | ＋ |  | C |
| 13SA002151 | 2013-3-17 | ST59 |  | ≥ 8 | >16 | >16 |  |  | ＋ |  | C |
| 13SA005001 | 2013-6-24 | ST59 |  | ≥ 8 | >16 | >16 |  |  | ＋ |  | C |
| 13SA009841 | 2013-7-3 | ST59 |  | ≥ 8 | >16 | >16 |  |  | ＋ |  | C |
| 13SA000691 | 2013-3-8 | ST59 |  | ≥ 8 | >16 | >16 |  |  | ＋ |  | C |
| 13SA007421 | 2013-7-2 | ST59 |  | ≥ 8 | >16 | >16 |  |  | ＋ |  | C |
| 13SA008251 | 2013-7-3 | ST59 |  | ≥ 8 | >16 | >16 |  |  | ＋ |  | C |
| 13SA000931 | 2013-8-4 | ST59 |  | ≥ 8 | >16 | 0.0625 |  |  | ＋ |  | C |
| 13SA004271 | 2013-7-3 | ST59 |  | ≥ 8 | >16 | 0.0625 |  |  | ＋ |  | C |
| 13SA005171 | 2013-7-2 | ST59 |  | ≥ 8 | >16 | 0.25 |  |  | ＋ |  | C |
| 12TA000451 | 2012-6-6 | ST59 |  | ≥ 8 | >16 | 0.25 |  |  | ＋ |  | C |
| 13SA006081 | 2013-3-8 | ST59 |  | ≥ 8 | >16 | 0.25 |  |  | ＋ |  | C |
| 13SA005291 | 2013-3-17 | ST59 |  | ≥ 8 | >16 | 0.5 |  |  | ＋ |  | C |
| 13SA006731 | 2013-6-9 | ST59 |  | ≥ 8 | >16 | 0.5 |  |  | ＋ |  | C |
| 13SA004131 | 2013-3-17 | ST59 |  | ≥ 8 | >16 | 0.5 |  |  | ＋ |  | C |
| 13SA001181 | 2013-3-17 | ST59 |  | ≥ 8 | >16 | 0.5 |  |  | ＋ |  | C |
| 13SA009931 | 2013-7-2 | ST59 |  | ≥ 8 | >16 | 0.5 |  |  | ＋ |  | C |
| 13SA004651 | 2013-7-3 | ST59 |  | ≥ 8 | 2 | 0.5 |  |  | ＋ |  | C |
| 13SA004521 | 2013-3-17 | ST59 |  | ≥ 8 | >16 | 2 |  |  | ＋ |  | C |
| 13SA006421 | 2013-6-9 | ST59 |  | ≥ 8 | >16 | 2 |  |  | ＋ |  | C |
| 13SA000731 | 2013-7-3 | ST59 |  | ≥ 8 | >16 | 4 |  |  | ＋ |  | C |
| 13SA002151 | 2013-3-17 | ST59 |  | ≥ 8 | >16 | 4 |  |  | ＋ |  | C |
| 13SA006161 | 2013-7-2 | ST59 |  | ≥ 8 | >16 | 16 |  |  | ＋ |  | C |
| 13SA008101 | 2013-7-3 | ST59 |  | ≥ 8 | >16 | >16 |  |  |  |  | C |
| 14SA003121 | 2014-6-5 | ST630 |  | ≥ 8 | >16 | 0.03125 |  |  |  | ＋ | I |
| 13SA002841 | 2013-3-17 | ST942 |  | ≥ 8 | >16 | >16 |  | ＋ |  |  | C |

**MLST**, Multilocus Sequence Typing; **MIC**, minimum inhibitory concentration; **MRSA**,

methicillin-resistant *Staphylococcus aureus*; **NT***,* not detected; **Ery**, Erythromycin; **Tel**,

Telithromycin; **Cet**, Cethromycin; **+**, positive; D-test: **C**, cMLSB (constitutive MLSB); **I**, iMLSB (inducible MLSB);

**Table S2**. The supplementary information of 124 MSSA in this study.

| **Isolates-ID** | **Isolated date** | **MLSTs** |  | **MIC (mg/L)** | | |  | ***ermA*** | ***ermB*** | ***ermC*** | **D-test** |
| --- | --- | --- | --- | --- | --- | --- | --- | --- | --- | --- | --- |
|  |  |  |  | **Ery** | **Tel** | **Cet** |  |  |  |  |  |
| 13SA001281 | 2013-7-3 | NT |  | ≥ 8 | >16 | >16 |  |  | + | + | C |
| 14SA003331 | 2014-6-8 | NT |  | ≥ 8 | 0.25 | 2 |  | ﹢ |  |  | I |
| 15SA002601 | 2015-4-13 | NT |  | ≥ 8 | 0.25 | 0.25 |  |  |  | ﹢ | I |
| 12TA000981 | 2012-7-23 | NT |  | ≥ 8 | 0.25 | 0.0625 |  |  | ﹢ | ﹢ | I |
| 13SA007431 | 2013-7-2 | NT |  | ≥ 8 | >16 | 0.0625 |  |  |  | ﹢ | I |
| 13SA003901 | 2013-3-17 | NT |  | ≥ 8 | >16 | 0.0625 |  |  |  | ﹢ | I |
| 15SA000251 | 2015-4-13 | NT |  | ≥ 8 | >16 | >16 |  |  |  | ﹢ | I |
| 13SA001831 | 2013-3-17 | NT |  | ≥ 8 | >16 | >16 |  |  |  | ﹢ | I |
| 13SA010771 | 2013-4-5 | NT |  | ≥ 8 | 8 | 0.0625 |  |  |  | ﹢ | C |
| 13SA010071 | 2013-7-3 | NT |  | ≥ 8 | >16 | >16 |  |  |  | ﹢ | C |
| 13SA001471 | 2013-7-2 | NT |  | ≥ 8 | >16 | 0.0625 |  |  | ﹢ |  | C |
| 15SA000791 | 2015-4-12 | ST1 |  | ≥ 8 | >16 | >16 |  |  |  | ﹢ | I |
| 13SA009061 | 2013-4-9 | ST1 |  | ≥ 8 | >16 | 0.03125 |  |  |  | ﹢ | I |
| 13SA008951 | 2013-4-9 | ST120 |  | ≥ 8 | >16 | 0.0625 |  |  |  | ﹢ | I |
| 13SA007831 | 2013-7-2 | ST120 |  | ≥ 8 | >16 | >16 |  |  |  | ﹢ | C |
| 13SA007941 | 2013-7-3 | ST120 |  | ≥ 8 | 2 | >16 |  |  |  | + | C |
| 13SA006711 | 2013-3-17 | ST120 |  | 0.5 | >16 | 0.0625 |  |  |  | ﹢ | C |
| 13SA009011 | 2013-1-23 | ST120 |  | ≥ 8 | >16 | 0.03125 |  |  |  | ﹢ | C |
| 13SA000061 | 2013-7-3 | ST120 |  | ≥ 8 | 8 | 0.03125 |  |  |  |  | C |
| 13SA006341 | 2013-7-2 | ST121 |  | ≥ 8 | >16 | 0.125 |  |  |  | ﹢ | C |
| 13SA002551 | 2013-7-7 | ST1281 |  | ≥ 8 | 16 | >16 |  |  |  | ﹢ | I |
| 13SA009581 | 2013-7-2 | ST15 |  | ≥ 8 | 4 | 0.5 |  |  |  | ﹢ | I |
| 13SA006981 | 2013-7-3 | ST188 |  | ≥ 8 | 0.25 | 0.25 |  |  |  | ﹢ | I |
| 13SA003071 | 2013-3-17 | ST188 |  | ≥ 8 | 1 | 0.25 |  |  |  | ﹢ | I |
| 13SA005651 | 2013-7-3 | ST1921 |  | ≥ 8 | 0.5 | 0.125 |  |  |  | ﹢ | I |
| 13SA006471 | 2013-7-3 | ST20 |  | ≥ 8 | 1 | 0.5 |  |  |  | ﹢ | I |
| 13TA000021 | 2013-1-23 | ST2144 |  | ≥ 8 | 0.25 | 0.25 |  | ﹢ |  |  | I |
| 15SA003221 | 2015-4-9 | ST217 |  | ≥ 8 | >16 | >16 |  |  |  | ﹢ | I |
| 13SA008481 | 2013-1-30 | ST217 |  | ≥ 8 | 0.5 | 0.0625 |  |  |  | ﹢ | I |
| 13SA007121 | 2013-7-3 | ST239 |  | ≥ 8 | 2 | 0.0625 |  | ＋ |  |  | C |
| 14SA003231 | 2014-6-8 | ST25 |  | ≥ 8 | 4 | 0.625 |  |  |  | ﹢ | I |
| 13SA007611 | 2013-7-2 | ST25 |  | ≥ 8 | 16 | 0.0625 |  |  |  | ﹢ | I |
| 13SA007971 | 2013-7-3 | ST25 |  | ≥ 8 | >16 | 0.0315 |  |  |  | ﹢ | I |
| 13SA009261 | 2013-7-2 | ST25 |  | ≥ 8 | >16 | >16 |  |  |  | + | C |
| 13SA005341 | 2013-7-3 | ST2797 |  | 4 | >16 | >16 |  |  |  | ﹢ | C |
| 13SA001951 | 2013-3-17 | ST2871 |  | ≥ 8 | 0.5 | 0.125 |  |  |  | ﹢ | I |
| 14SA000031 | 2014-6-9 | ST30 |  | ≥ 8 | 0.25 | 0.25 |  | ﹢ |  |  | I |
| 13SA004591 | 2013-7-3 | ST30 |  | 1 | 0.125 | 0.5 |  | ﹢ |  |  | I |
| 13SA001461 | 2013-3-17 | ST30 |  | ≥ 8 | 0.5 | 0.0625 |  | ﹢ |  |  | I |
| 13SA004541 | 2013-1-23 | ST30 |  | ≥ 8 | 0.25 | 0.0625 |  | ﹢ |  |  | I |
| 13SA007241 | 2013-7-3 | ST363 |  | ≥ 8 | 2 | 0.0625 |  |  |  | ﹢ | I |
| 13SA002321 | 2013-7-8 | ST398 |  | ≥ 8 | >16 | >16 |  |  |  | ﹢ | I |
| 13SA010091 | 2013-7-3 | ST398 |  | ≥ 8 | >16 | >16 |  |  |  | ﹢ | I |
| 14SA000681 | 2014-7-6 | ST398 |  | ≥ 8 | 4 | 0.125 |  | ﹢ |  |  | I |
| 14SA001791 | 2014-7-7 | ST398 |  | ≥ 8 | 2 | 0.25 |  |  | ﹢ |  | C |
| 13SA000111 | 2013-1-30 | ST398 |  | ≥ 8 | >16 | 0.0625 |  |  | ﹢ |  | C |
| 14SA002171 | 2014-6-7 | ST398 |  | ≥ 8 | >16 | 0.0625 |  |  | ﹢ |  | C |
| 12TA000111 | 2012-7-23 | ST398 |  | ≥ 8 | >16 | >16 |  |  |  | ﹢ | C |
| 13SA002561 | 2013-3-17 | ST398 |  | ≥ 8 | >16 | >16 |  |  | ﹢ |  | C |
| 13SA009211 | 2013-7-2 | ST398 |  | ≥ 8 | >16 | >16 |  |  | ﹢ |  | C |
| 14SA003441 | 2014-9-6 | ST398 |  | ≥ 8 | >16 | 0.25 |  |  | ﹢ |  | C |
| 15SA003051 | 2015-8-5 | ST398 |  | ≥ 8 | 0.5 | 0.0625 |  |  | ﹢ |  | C |
| 13SA009921 | 2013-5-2 | ST398 |  | ≥ 8 | >16 | 0.03125 |  |  | ﹢ |  | C |
| 12TA001171 | 2012-7-23 | ST5 |  | ≥ 8 | >16 | 0.0625 |  |  |  | ﹢ | I |
| 15SA000831 | 2015-8-1 | ST5 |  | ≥ 8 | >16 | 0.0625 |  |  |  | ﹢ | I |
| 13SA003801 | 2013-3-17 | ST5 |  | ≥ 8 | >16 | >16 |  |  |  | ﹢ | I |
| 13SA008591 | 2013-7-2 | ST5 |  | ≥ 8 | >16 | 0.0625 |  |  |  | ﹢ | I |
| 13SA010991 | 2013-7-2 | ST59 |  | ≥ 8 | >16 | >16 |  |  |  | ﹢ | I |
| 16SA000091 | 2016-6-2 | ST59 |  | ≥ 8 | 8 | 4 |  |  | + |  | C |
| 15SA000071 | 2015-8-5 | ST59 |  | ≥ 8 | >16 | 0.5 |  |  | + |  | C |
| 13SA001251 | 2013-3-8 | ST59 |  | ≥ 8 | >16 | 0.25 |  |  | ﹢ |  | C |
| 13SA004701 | 2013-3-17 | ST59 |  | ≥ 8 | >16 | >16 |  |  | + |  | C |
| 13SA004931 | 2013-7-2 | ST59 |  | ≥ 8 | >16 | >16 |  |  | + |  | C |
| 13SA002071 | 2013-7-3 | ST59 |  | ≥ 8 | >16 | >16 |  |  | ﹢ |  | C |
| 13SA008461 | 2013-3-16 | ST59 |  | ≥ 8 | >16 | >16 |  |  | ﹢ |  | C |
| 14SA002751 | 2014-6-5 | ST59 |  | ≥ 8 | >16 | >16 |  |  | ﹢ |  | C |
| 13SA006611 | 2013-7-3 | ST59 |  | ≥ 8 | >16 | >16 |  |  | ﹢ |  | C |
| 15SA003131 | 2015-8-3 | ST59 |  | ≥ 8 | 0.25 | >16 |  |  | + |  | C |
| 16SA000121 | 2016-6-2 | ST59 |  | ≥ 8 | >16 | >16 |  |  | ﹢ |  | C |
| 13SA010561 | 2013-7-3 | ST59 |  | ≥ 8 | >16 | >16 |  |  |  |  | C |
| 13SA005061 | 2013-6-24 | ST59 |  | ≥ 8 | >16 | >16 |  |  |  |  | C |
| 13SA007841 | 2013-7-2 | ST59 |  | ≥ 8 | >16 | 1 |  |  | ﹢ |  | C |
| 14SA001751 | 2014-6-8 | ST59 |  | ≥ 8 | >16 | 0.5 |  |  | ﹢ |  | C |
| 15SA001301 | 2015-8-7 | ST59 |  | ≥ 8 | 16 | 0.5 |  |  | ﹢ |  | C |
| 13SA003921 | 2013-7-3 | ST59 |  | ≥ 8 | >16 | 0.25 |  |  | ﹢ |  | C |
| 14SA000401 | 2014-6-3 | ST59 |  | ≥ 8 | >16 | 0.25 |  |  | ﹢ |  | C |
| 13SA000821 | 2013-3-17 | ST59 |  | ≥ 8 | >16 | 0.125 |  |  | ﹢ |  | C |
| 15SA000671 | 2015-9-2 | ST6 |  | ≥ 8 | >16 | 2 |  |  |  | ﹢ | I |
| 13SA006561 | 2013-7-3 | ST6 |  | ≥ 8 | >16 | >16 |  |  |  | ﹢ | I |
| 13SA008061 | 2013-3-6 | ST6 |  | ≥ 8 | >16 | 0.125 |  |  |  | ﹢ | I |
| 13SA004081 | 2013-6-6 | ST630 |  | ≥ 8 | >16 | >16 |  |  |  | ﹢ | I |
| 13SA005541 | 2013-7-2 | ST630 |  | ≥ 8 | 0.25 | 0.0625 |  | ﹢ |  |  | I |
| 13SA001111 | 2013-3-8 | ST633 |  | ≥ 8 | >16 | >16 |  |  | ﹢ |  | C |
| 14SA001621 | 2014-1-6 | ST633 |  | 4 | >16 | >16 |  |  | ﹢ |  | C |
| 13SA006271 | 2013-7-3 | ST633 |  | ≥ 8 | >16 | 4 |  |  | ﹢ |  | C |
| 13SA001171 | 2013-3-17 | ST633 |  | ≥ 8 | >16 | 0.5 |  |  | ﹢ |  | C |
| 12TA000521 | 2012-7-23 | ST7 |  | ≥ 8 | 0.25 | 16 |  |  |  | ﹢ | I |
| 15SA003411 | 2015-8-2 | ST7 |  | ≥ 8 | >16 | 0.5 |  |  |  | ﹢ | I |
| 13SA005951 | 2013-7-3 | ST7 |  | ≥ 8 | 0.125 | 0.25 |  |  |  | ﹢ | I |
| 15SA002201 | 2015-9-2 | ST7 |  | ≥ 8 | 0.125 | 0.25 |  |  |  | ﹢ | I |
| 13SA007191 | 2013-7-3 | ST7 |  | ≥ 8 | 2 | 0.25 |  |  |  | ﹢ | I |
| 13SA009021 | 2013-7-2 | ST7 |  | ≥ 8 | >16 | 0.0625 |  |  |  | ﹢ | I |
| 13SA008611 | 2013-7-2 | ST7 |  | ≥ 8 | >16 | 0.0625 |  |  |  | ﹢ | I |
| 13SA009221 | 2013-7-2 | ST7 |  | ≥ 8 | 4 | 0.0625 |  |  |  | ﹢ | I |
| 14SA001291 | 2014-1-6 | ST7 |  | ≥ 8 | >16 | 0.0625 |  |  |  | ﹢ | I |
| 15SA000911 | 2015-8-3 | ST7 |  | ≥ 8 | >16 | 0.0625 |  |  |  | ﹢ | I |
| 13SA007361 | 2013-7-2 | ST7 |  | ≥ 8 | 16 | 0.0625 |  |  |  | ﹢ | I |
| 15SA001991 | 2015-8-5 | ST7 |  | ≥ 8 | >16 | 0.03125 |  |  |  | ﹢ | I |
| 15SA000431 | 2015-5-4 | ST7 |  | ＞8 | >16 | >16 |  |  |  | ﹢ | I |
| 13SA001901 | 2013-3-17 | ST7 |  | ≥ 8 | >16 | >16 |  |  |  | ﹢ | I |
| 13SA009121 | 2013-7-2 | ST7 |  | ≥ 8 | >16 | >16 |  |  |  | ﹢ | I |
| 13SA001871 | 2013-3-17 | ST7 |  | ≥ 8 | >16 | >16 |  |  |  | ﹢ | I |
| 13SA006721 | 2013-7-3 | ST7 |  | ≥ 8 | >16 | >16 |  |  |  | ﹢ | I |
| 13SA007081 | 2013-7-3 | ST7 |  | ≥ 8 | >16 | >16 |  |  |  | ﹢ | I |
| 14SA000341 | 2014-2-6 | ST7 |  | ≥ 8 | >16 | >16 |  |  |  | ﹢ | I |
| 13SA009231 | 2013-7-3 | ST7 |  | ≥ 8 | >16 | 0.03125 |  |  |  | ﹢ | I |
| 13SA004231 | 2013-7-2 | ST7 |  | ≥ 8 | >16 | >16 |  |  | ﹢ |  | C |
| 13SA002091 | 2013-3-17 | ST7 |  | ≥ 8 | >16 | >16 |  |  | ﹢ |  | C |
| 13SA002011 | 2013-3-15 | ST7 |  | ≥ 8 | >16 | 0.5 |  |  | ﹢ |  | C |
| 14SA000751 | 2014-6-6 | ST7 |  | ≥ 8 | >16 | 0.5 |  |  | ﹢ |  | C |
| 15SA001981 | 2015-8-3 | ST7 |  | ≥ 8 | >16 | 0.5 |  |  | ﹢ |  | C |
| 13SA006571 | 2013-7-2 | ST7 |  | ≥ 8 | >16 | 0.125 |  |  | ﹢ |  | C |
| 13SA001061 | 2013-3-17 | ST7 |  | ≥ 8 | >16 | 0.03125 |  |  | ﹢ |  | C |
| 13SA008161 | 2013-7-3 | ST8 |  | ≥ 8 | >16 | 0.0625 |  |  |  | ﹢ | I |
| 13SA008271 | 2013-7-3 | ST88 |  | ≥ 8 | 2 | 0.0625 |  |  |  | ﹢ | I |
| 13SA001341 | 2013-3-13 | ST88 |  | ≥ 8 | >16 | >16 |  |  |  | ﹢ | I |
| 13SA002161 | 2013-3-17 | ST88 |  | ≥ 8 | 1 | 0.0625 |  |  |  | ﹢ | I |
| 13SA009101 | 2013-7-3 | ST88 |  | ≥ 8 | 8 | 0.03125 |  |  |  | ﹢ | I |
| 13SA006061 | 2013-7-2 | ST88 |  | ≥ 8 | 0.25 | 0.5 |  |  | + |  | C |
| 12TA000031 | 2012-7-23 | ST88 |  | ≥ 8 | >16 | >16 |  |  | + |  | C |
| 13SA011081 | 2013-7-2 | ST88 |  | ≥ 8 | 4 | 0.5 |  |  | + |  | C |
| 13SA006811 | 2013-7-3 | ST965 |  | ≥ 8 | 0.125 | 0.25 |  |  |  | ﹢ | I |
| 13SA005701 | 2013-7-3 | ST965 |  | ≥ 8 | 2 | 0.0625 |  |  |  | ﹢ | I |
| 13SA002431 | 2013-3-8 | ST965 |  | ≥ 8 | >16 | 0.5 |  |  |  | + | I |

**MLST**, Multilocus Sequence Typing; **MIC**, minimum inhibitory concentration; **MSSA**,

methicillin-sensitive *Staphylococcus aureus*; **NT***,* not detected; **Ery**, Erythromycin; **Tel**,

Telithromycin; **Cet**, Cethromycin; **+**, positive; D-test: **C**, cMLSB (constitutive MLSB); **I**, iMLSB (inducible MLSB);

**Table S3**. PCR primers used for *S. aureus* MLST gene diversity determination.

| **Target** | **Primer** | **Primer Sequence(5’-3’)** | **Amplicon size (bp)** | **Ref.** |
| --- | --- | --- | --- | --- |
| ***arcC*** | *arcC*-F | TTGATTCACCAGCGCGTATTGTC | 456bp | Enright *et al* , 2000 |
|  | *arcC*-R | AGGTATCTGCTTCAATCAGCG |  |  |
| ***aroE*** | *aroE*-F | ATCGGAAATCCTATTTCACATTC | 456bp | Enright *et al* , 2000 |
|  | *aroE*-R | GGTGTTGTATTAATAACGATATC |  |  |
| ***glpF*** | *glpF*-F | CTAGGAACTGCAATCTTAATCC | 465bp | Enright *et al* , 2000 |
|  | *glpF*-R | TGGTAAAATCGCATGTCCAATFC |  |  |
| ***gmk*** | *gmk*-F | ATCGTTTTATCGGGACCATC | 429bp | Enright *et al* , 2000 |
|  | *gmk*-R | TCATTAACTACAACGTAATCGTA |  |  |
| ***pta*** | *pta*-F | GTTAAAATCGTATTACCTGAAGG | 474bp | Enright *et al* , 2000 |
|  | *pta*-R | GACCCTTTTGTTGAAAAGCTTAA |  |  |
| ***tpi*** | *tpi*-F | TCGTTCATTCTGAACGTCGTGAA | 402bp | Enright *et al* , 2000 |
|  | *tpi*-R | TTTGCACCTTCTAACAATTGTAC |  |  |
| ***yqiL*** | yqil-F | CAGCATACAGGACACCTATTGGC | 516bp | Enright *et al* , 2000 |
|  | yqil-R | CGTTGAGGAATCGATACTGGAAC |  |  |

**Table S4**. PCR primers used for *S. aureus* *erm* genes detection.

| **Target** | **Primer** | **Primer Sequence(5’-3’)** | **Amplicon size (bp)** | **Ref.** |
| --- | --- | --- | --- | --- |
| ***ermA*** | *ermA*-F | TCTAAAAAGCATGTAAAAGAAA | 553bp | Schwarz *et al* , 2002 |
|  | *ermA*-R | CGATACTTTTTGTAGTCCTTC |  |  |
| ***ermB*** | *ermB*-F | CCGTTTACGAAATTGGAACAGGTAAAGGGC | 359bp | Lina *et al* , 1999 |
|  | *ermB*-R | GAATCGAGACTTGAGTGTGC |  |  |
| ***ermC*** | *ermC*-F | GCTAATATTGTTTAAATCGTCAATTCC | 460bp | Schwarz *et al* , 2002 |
|  | *ermC*-R | GGATCAGGAAAAGGACATTTTAC |  |  |
